# Supplementary material for: Inula britannica Inhibits Adipogenesis of 3T3-L1 Preadipocytes via Modulation of Mitotic Clonal Expansion Involving ERK 1/2 and Akt Signaling Pathways
Source: Nutrients. 2020 Oct 3;12(10):3037. doi: 10.3390/nu12103037 (PMC7599673; doi:10.3390/nu12103037)
Supplement: Supplementary file 1 [file nutrients-12-03037-s001.pdf]

Table S1. Primer sequences employed in quantitative real-time PCR.

Gene	Primer sequence (5′→3 ′)
Fasn	Sense: ACCTGGTAGACCACTGCATTGAC<br>Antisense: CCTGATGAAACGACACATTCTCA
Fabp4	Sense: ACACCGAGATTTCCTTCAAACTG<br>Antisense: CCATCTAGGGTTATGATGCTCTTCA
Plin1	Sense: TACCCTCCAGAAAAGATCGC<br>Antisense: CTACCACCTTCTCGATGCTT
Scd1	Sense: CTGCCCCTGCGGATCTT<br>Antisense: GCCCATTCGTACACGTCATTCT
Pparg	Sense: CACAAGAGCTGACCAATGGT<br>Antisense: GATCGCACTTTGGTATTCTTGGA
Cebpa	Sense: GCGCAAGAGCCGAGATAAAG<br>Antisense: CGGTCATTGTCACTGGTCAACT
Srebp1c	Sense: CCCTACCGGTCTTCTATCAATGA<br>Antisense: GCAGATTTATTCAGCTTTGCTTCA
Adipoq	Sense: GATGGCACTCCTGGAGAGAA<br>Antisense: TCTCCAGGCTCTCCTTTCCT
Tbp	Sense: ACCCTTCACCAATGACTCCTATG<br>Antisense: TGACTGCAGCAAATCGCTTGG

Fasn, fatty acid synthase; *Fabp4*, fatty acid binding protein 4; *Plin1*, perilipin 1; *Scd1*, stearoyl-CoA desaturase-1; *Pparg*, Peroxisome proliferator-activated receptor-γ, *Cebpa*, CCAAT/enhancer-binding protein-α; *Srebp1c*, sterol regulatory element-binding protein-1c; *Adipoq*, adiponectin; *Tbp*, TATA box-binding protein.

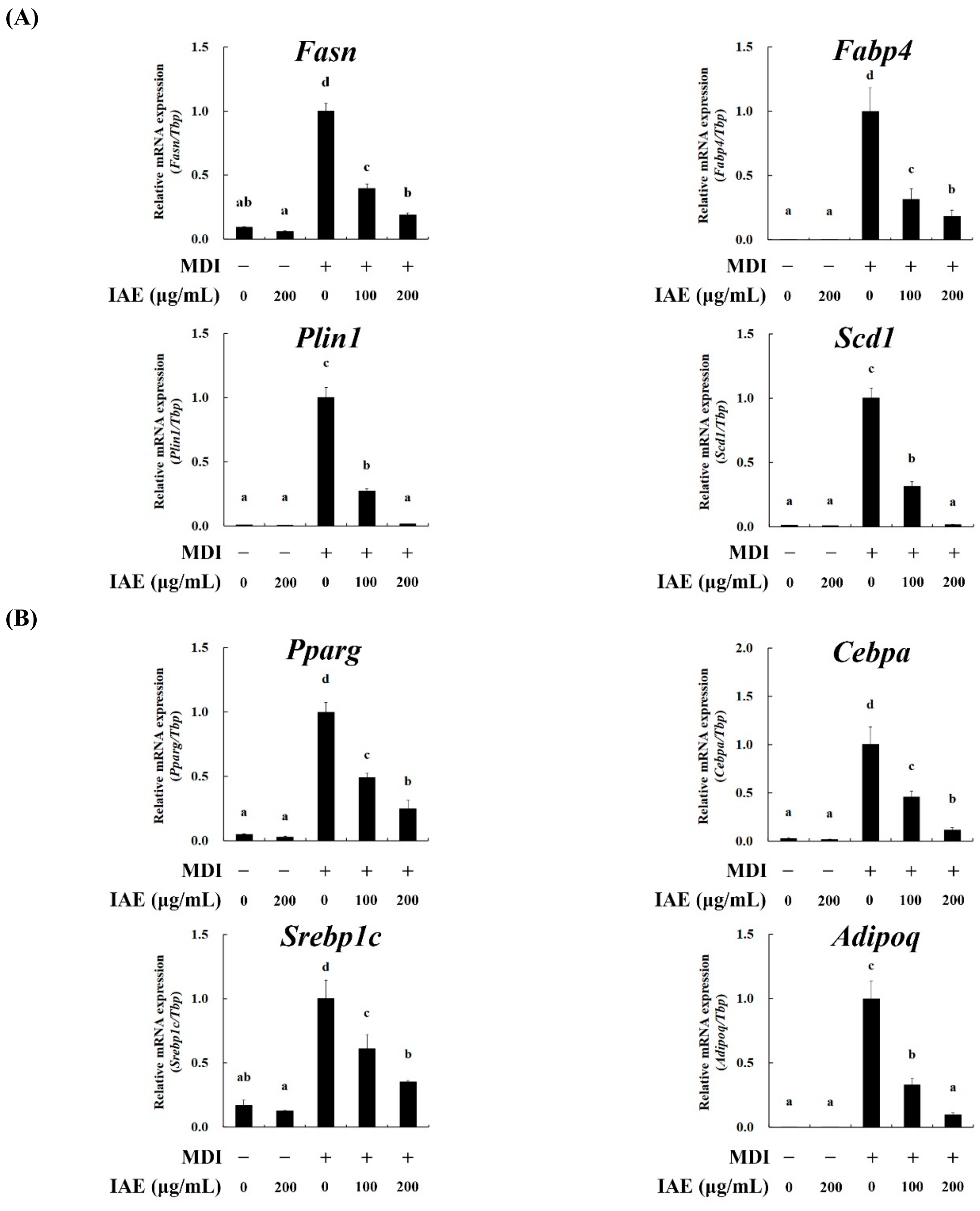

Figure S1. *Inula britannica* flower aqeuous extract (IAE) inhibits adipogenesis of 3T3-L1 preadipocytes via modulating the transcriptional expression. Cells were differentiated for 8 days upon MDI stimulation in the presence of the indicated concentrations of IAE. The mRNA expression of adipogenesis-associated biomarkers was evaluated by using qRT-PCR. Relative mRNA expression levels of each target genes were assessed following normalization with TATA box binding protein (*Tbp*). (A) Relative mRNA expression levels of lipogenesis-associated biomarkers. (B) Relative mRNA expression levels of adipogenesis-associated biomarkers. The data are presented as mean ± standard deviation. Values labeled with different letters are significantly different ($p < 0.05$). Differences among the multiple groups were determined based on one-tailed one-way analysis of variance, followed by Tukey's post hoc test.

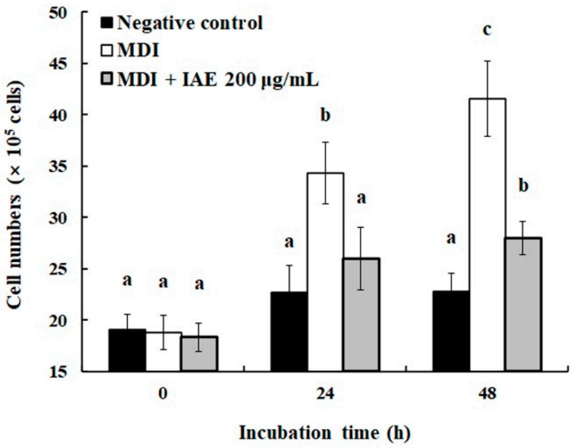

Figure S2. *Inula britannica* flower aqeuous extract (IAE) inhibits proliferation of 3T3-L1 preadipocytes during the MDI-induced MCE development. Two-day-post-confluent 3T3-L1 preadipocytes induced to undergo mitotic clonal expansion with MDI in presence of the indicated concentration of IAE. Viable cell numbers were calculated with trypan blue assay upon 0, 24, and 48 h of differentiation. The data are presented as mean ± standard deviation. Values labeled with different letters are significantly different ($p < 0.05$). Differences among the multiple groups were determined based on one-tailed one-way analysis of variance, followed by Tukey's post hoc test.

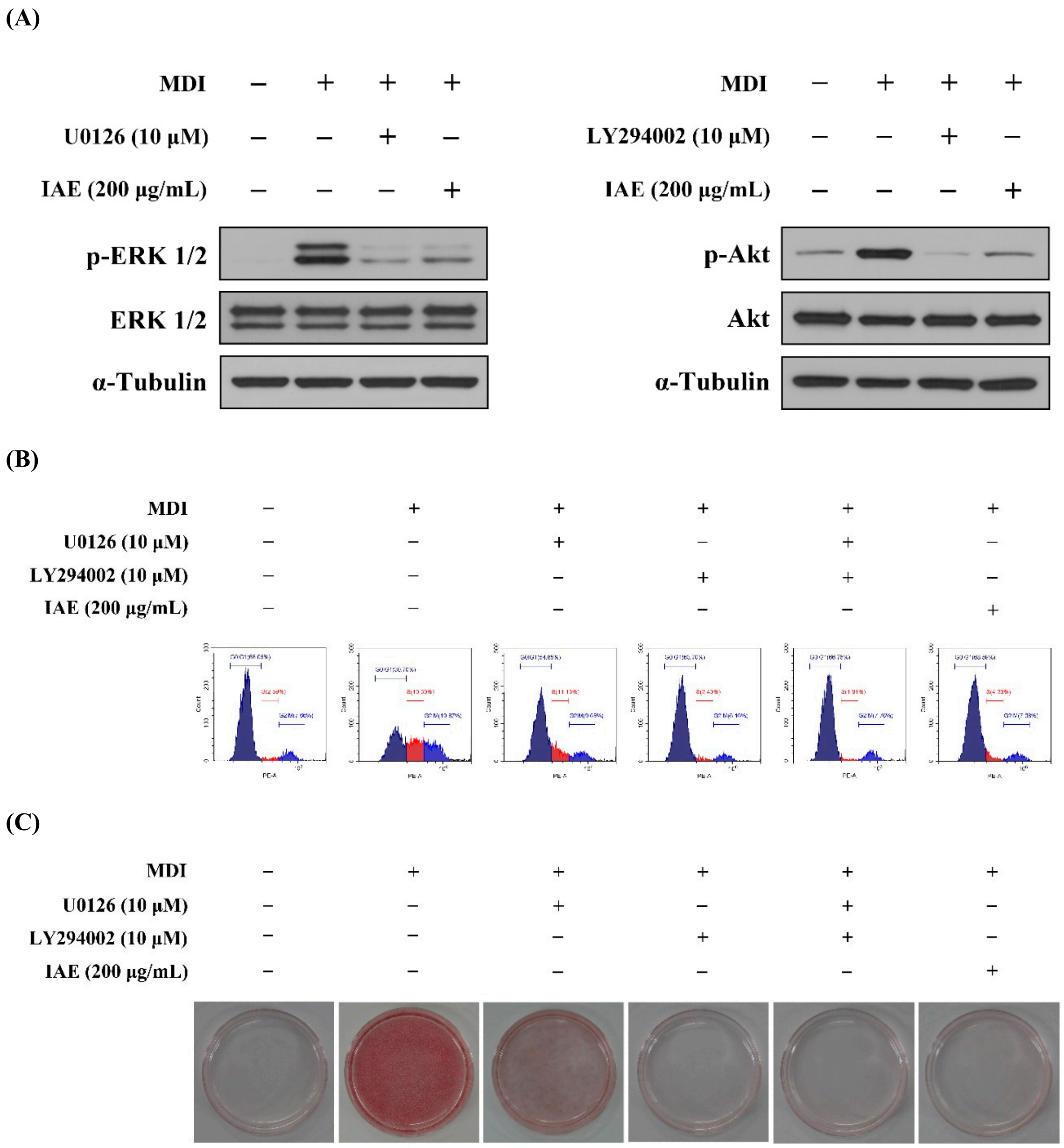

Figure S3. *Inula britannica* flower aqueous extract (IAE) inhibits MDI-induced adipogenesis of 3T3-L1 preadipocytes by regulating the ERK 1/2 and Akt signaling pathways. Two-day-post-confluent 3T3-L1 preadipocytes treated with MDI in the presence of IAE or specific inhibitors. The specific inhibitors, U0126 and LY294002, were treated 1 h prior to stimulation of MDI. The phosphorylation of MEK-1 (Ser217/221), ERK 1/2 (Thr202/Tyr204), and Akt (Ser473) was examined to evaluate activation of these proteins. (A) After 30 min of MDI stimulation, the phosphorylation of ERK 1/2 and Akt was assessed using western blotting. (B) After 16 h of MDI stimulation, the effect of ERK 1/2 and Akt signaling pathways on cell cycle progression was examined using FACS. (C) The effect of ERK 1/2 and Akt signaling pathways on accumulation of intracellular lipids was assessed using Oil Red O staining on day 8.
